# 

**Published:** 1896-01-11

**Authors:** 


**Notices of Births, Deaths, and Marriages** are inserted in this column at a charge of 2s. 6d. for an announcement not exceeding four lines.

**Notice to Advertisers and Subscribers.**

All Advertisements, Orders for Copies of The Hospital and Business Communications should be addressed to **THE MANAGER, (Not to The Editor),** The Hospital, 428, Strand, London, W.C.

The Cost of Subscription, post free, is as follows:—Per week, 2½d.; per quarter, 2s. 8d.; per half-year, 5s. 3d.; per annum, 10s. 6d. Foreign:—Per Annum, 15s. 2d.; payable, in all cases, in advance.

**NOTICE TO CORRESPONDENTS.**

All MS., Letters, Books for Review, and other matters intended for the Editor should be addressed The Editor, The Lodge, Porchester Square, London, W.

The Editor cannot undertake to return rejected MS., even when accompanied by stamped directed envelope.

**"Burdett's Hospital Annual," 1895.**

Sixth year of publication. 950 pp. Scarlet cloth, gilt lettered. Price 5s. A most complete guide to British, Colonial, Indian, and American Hospitals, Dispensaries, Nursing and Convalescent Institutions, and Asylums. A few complete sets of the preceding five issues of the "Annual" may still be obtained on application to the Publishers, The Scientific Press (Limited), 428, Strand, London, W.C.

**NOTICE.—Vol. XVIII. of "The Hospital" (April to September, 1895), in handsome cloth binding, is now on sale at the Office of this Journal. Price 7s. 6d. Cases for binding the Numbers contained in this Volume, 1s. 6d. Index to Vol. XVIII., 2½d., post free.**

**The Monthly Part for January is now ready, price 6d., by post, 8d.**

### SCRAPS AND GLEANINGS.

About £13,000 is being expended by the Fulham Union in addition to their infirmary.

The Fishmongers' Company have forwarded a donation of £105 to the Poplar Hospital for Accidents.

Miss Isabella Bird Bishop is about to build a hospital for women in Korea at her personal expense.

Over £1,000 has been subscribed this year to the Burton Infirmary by the working people of the locality.

The Princess Louise has sent £25 to the Grosvenor Hospital for Women and Children at Westminster.

A ball in aid of the East London Hospital for Children will take place on January 14th at the Portman Rooms.

The fifth annual ball for the benefit of the Bingley Cottage Hospital has been held at the Victoria Hall, Bingley, Yorks.

A munificent donation of £2,000 has been received from Mr. John M. Keiller by the directors of the Dundee Royal Infirmary.

The Saddlers' Company have made grants out of their corporate funds to hospitals and other public institutions exceeding £1,300.

The Southampton Hospital Saturday and Sunday Fund this year resulted in over £1,000 being handed over to the local hospitals.

The Duchess of Saxe-Coburg and Gotha forwarded a gift of toys and books for the inmates of the Victoria Hospital for Children.

The annual address on behalf of the Bradford Joint Hospital Board was given by the Bishop of Richmond, at St. George's Hall, Bradford.

Lord Iveagh has sent a donation of £200 to the governors of the Rotunda Hospital, Dublin, towards paying off the debt on the new wing.

The project of erecting an isolation hospital for North Ormesby is shortly to be entered into. The scheme will possibly provide for a con joint fever hospital.

Princess Louise (Marchioness of Lorne) has forwarded four framed pictures to ornament the wards of the Victoria Hospital for Children, of which H.R.H. is patron.

The estimated cost of the additions and alterations to the Guest Hospital, Dudley, is about £2,000, towards which donations received and promised amount to £1,350.

The Royal Hospital for Diseases of the Chest is the oldest hospital for this speciality in Europe. Funds are greatly needed. The income—of a fluctuating order—does not reach, yearly, half what is required for its maintenance.

The working people of Lancaster have shown sympathy towards the local infirmary in a very practical manner; and their annual subscription and collection list this year exhibits a still greater increase of figures than previously.

The president of the Evelina Hospital, Southwark, having purchased a plot of land adjoining the hospital on the south side, it is proposed to extend the building so that 34 more children can find accommodation. This will, in all, provide for the reception of 100 little patients.

At the annual meeting of the Dawlish Dispensary Committee the medical report showed that the year on the whole had been a healthy one, and no deaths had occurred during that time among the 246 sick persons under treatment. A small adverse balance remains on the accounts of the dispensary.

The annual report of the East Suffolk and Ipswich Hospital is of a satisfactory nature. Subscriptions for the year show some increase, whilst the expenditure for the same period has been somewhat less. Mr. Herbert Mason has offered a donation of £50 towards the cost of a new boiler and heating apparatus, which is a somewhat heavy item on the accounts.

Last year there were 11,057 patients at the Royal Westminster Ophthalmic Hospital, of which number 432 were in-patients, and since its foundation in 1816 up to last year no fewer than 427,789 patients have been attended to. The committee set themselves a task of raising £50,000 to rebuild the hospital on a larger site, and thus enabling them to give accommodation for 70 more beds.

The claims of the Children's Aid Society have again been brought before the notice of the charitable public. The work effected by its members commenced in 1856, since when 4,925 children have been placed in schools or homes, and 24,475 little ones otherwise aided. Payments have been made to homes for the maintenance of poor children in all parts of the kingdom amounting to £48,065.

### THE STUDENT OF MEDICINE.

### APPOINTMENTS.

D. T. Belding, M.R.C.S., L.R.C.P.Lond., appointed Medical Officer of Health to the East Dereham Urban District Council. R. W. Brimacombe, M.R.C.S., L.R.C.P., appointed Medical Officer for the Warmley Out-relief District of the Kingswood Union. K. C. Chetwood-Aiken, M.B.Aberd., appointed House Surgeon to the Central London Ophthalmic Hospital. Dr. J. Clark, appointed Medical Officer for the Fourth District of the Barnsley Union. Dr. J. W. Cook, appointed Medical Officer of Health to the Urban District Council of Walton-on-the-Naze. E. J. Cross, M.R.C.S., L.R.C.P.Lond., appointed Medical Officer for the Second District of the St. Neots Union. M. G. Dundas, M.R C.S.Eng., appointed Medical Officer for the East Dereham District of the Mitford and Launditch Union. T. Elliott, B.A.Dub., M.D., L.R.C.S.I., appointed Medical Officer for the No. 1 District of the Tonbridge Union. D. P. Fraser, M.D., appointed Medical Officer of Health for the Borough of Conway. P. Fraser, M.D.Glasg., L.R.C.S.Edin., appointed Medical Officer of Health for the Borough of Conway. G. E. Fryer, M.R.C.S., L.R.C.P.Lond., appointed Assistant Medical Officer to the Manchester Royal Infirmary. T. W. H. Garstang, B.A.Oxon., M.R.C.S.Eng., appointed Medical Officer of Health to the Bucklow Rural District Council. Mr. T. A. Greene, appointed Assistant Medical Officer to the Clare Lunatic Asylum. G. E. Hale, M.B.Camb., B.C., M.R.C.S., appointed Medical Officer for the Eton District of the Eton Union. J. T. Haworth, L.R.C.P., L.R.C.S.Edin., appointed Medical Officer of Health for the Filey Urban District. J. C. Heaven, L.R.C.P.Lond., M.R.C.S.Eng., appointed temporarily Medical Officer of Health to the Keynsham District Council. F. Hunton, M.D.Durh., M.B., B.S., appointed Medical Officer for the Stockton District and the Workhouse of the Stockton Union. Dr. T. H. Jones, appointed Medical Officer

**THE STUDENT OF MEDICINE**—*continued.*

of Health to the Newport (Mon.) Port Sanitary Authority. J. P. Lowe, M.B., Ch.B., appointed Assistant Medical Officer of the Monsall Fever Hospital, Manchester. R. G. McKerron, M.A.Abard., M.B, C.M., appointed Junior Physician to the Children's Hospital, Aberdeen. J. A. Manton, L.R.C.P.Lond., M.R.C.S., appointed Medical Officer to the Sheffield Post Office. Dr. M. J. Mergler, appointed Gynæcologist and Head Physician and Surgeon to the Mary Thompson Hospital for Women and Children, Chicago. Dr. W. F. Miller, appointed Medical Officer for the Alwinton District of the Rothbury Union. Dr. W. Oliver, appointed District Medical Officer of the Saddleworth Union. A R. Rackham, L.R.C.P. Edin , M.R.C.S Eng., appointed Medic l Officer of the Workhouse of the Milford and Launditch Union. T. W. Richardson, M.R.C.S., L.S.A., appointed Medical Officer of Health to the Erpingham Rural District. A. H. Roberts, L.R.C.P.Lond., M.R.C.S., appointed Medical Officer of Health for the Malling Rural District. A. T. Smith, M.B., C.M., appointed Medical Officer for the Orpington (No. 3) District of the Bromley Union. R. Smith, M.R.C.S., L.R.C.P., appointed House Surgeon to the West Norfolk and Lynn Hospital, King's Lynn. T. A. Somerville, L.R.C.P.Edin., L.M., M.R.C.S , appointed Medical Officer of Health to the Wilmslow Urban District Council. Dr. S. Spicer, appointed an Honorary Physician to the Royal Society of Musicians of Great Britain. G. E. Stewart, M.B., C.M.Edin., appointed Assistant House Surgeon to the Scarborough Hospital and Dispensary. W. E. Sturgess-Jones, M.R.C.S., L.R.C.P., appointed Resident Medical Officer to the Peruvian Corporation. H. C. Thompson, M.D.Lond., M.R C.P., appointed Medica Registrar to the Middlesex Hospital. J. H. Tonking, M.B. L.R.C.P.Lond., M.R.C.S., appointed Medical Officer for the Camborne District of the Redruth Union. Dr. Wickham, appointed Medical Officer for the Fourth District of the East Ashford Union. Dr. J. C. V. Wilkins, appointed Medical Officer for the Third District of the Liskeard Union.

### VACANCIES.

The following vacancies are announced this week, the amount of ·alary in each case being given after the name of the institution :—

#### Resident Medical Officers.

Birmingham General Dispensary.—Resident Surgeon; doubly qualified. Salary £150 per annum, with allowance of £30 per annum for cab hire, and furnished rooms, fire, lights, and attendance. Applications to Alex. Forrest, Secretary, by January 20th.

County Asylum, Upton, near Chester.—Junior Assistant Medical Officer: unmarried, and not more than 30 years of age. Salary £120 per annum, with board (no liquors), lodging, and washing. Applications to Dr. Lawrence, County Asylum, Chester, by January 15th.

County Borough of Cardiff.—Resident Medical Officer for the Borough Hospital for Infectious Diseases: unmarried. Appointment for one year. Salary £50 per annum, with board (without stimulants), and residence in the hospital. Applications to Dr. Walford, Medical Officer of Health, Town Hall, Cardiff, by January 18th.

General Infirmary and Dispensary, Doncaster.—Indoor Dispenser and Assi tant to House Surgeon. No salary, but board, lodging, and washing provided. Applications to Joseph Clark, Honorary Secretary, by January 15th.

Parish of Ronsay and Egelshay, Orkney.—Resident Medical Officer. Salary £51 per annum. Applications to Inspector of Poor, Ronsay, Orkney, by January 15th.

Royal Albert Hospital, Devonport.—Assistant House Surgeon for six months. No salary. Board, residence, and washing provided. Applications to the Secretary, Medical Committee, by January 11th.

Scottish Prison Service.—Resident Medical Officer. Salary £250, with a house. Applications to the Secretary of the Prison Commission for Scotland, 6, Rutland Square, Edinburgh, by January 31st.

Victoria Hospital for Sick Children, Queen's Road, Chelsea, S.W.—House Surgeon to the In-Patients; appointment for twelve months; must be F. or M.R.C.S.Eng. Honorarium £50 per annum, with board and lodging in the hospital. Also House Physician to the In-Patients; appointment for eight months. Honorarium at the rate of £50 per annum, with board and lodging in the hospital. Applications to the Secretary by January 11th.

#### Non-Resident Medical Officers.

Central London Throat, Nose, and Ear Hospital, Gray's Inn Road, W.C. —Assistant Registrars. Applications to Richard Kershaw, Secretary, by January 11th.

City Orthopædic Hospital, Hatton Garden.—Assistant Surgeon; must be F. or M.R.C.S.Eng Applications to the Committee by January 17th.

Edmonton Union.—Non-resident Medical Officer for the Chase Farm Schools, Enfield. Salary £80 per annum. Applications, on forms provided, to be sent to Francis Shelton, Solicitor and Clerk, The Grange, White Hart Lane, Tottenham, by January 14th.

Hospital for Women, Soho Square, W.—Registrar; must be graduate in medicine of some recognised university. Appointment for twelve months. Honorarium 25 guineas. Applications to David Cannon, Secretary, by January 14th.

University of London.—Registrarship. Salary commences at £800, and rising by annual increments to £1,000 per annum Applications to Arthur Milman, M.A., LL.D., Registrar, University of London, Burlington Gardens, W., by January 25th.

West-end Hospital for Diseases of the Nervous System, Welbeck Street, W.—Physician. Must be Fellow or Member of the Royal College of Physicians of London, and graduate of a British University. Applications to H. Heckstall-Smith, Secretary, at the Hospital, by January 13th.

### "THE HOSPITAL"

AN INDEPENDENT JOURNAL OF
**The Medical Sciences**
AND
**Hospital Administration,**
WITH WHICH IS ISSUED WEEKLY
A SPECIAL
**SUPPLEMENT FOR NURSES.**

PUBLISHED EVERY SATURDAY.

PRICE TWOPENCE.

Matrons, Sisters, or Nurses will find in THE HOSPITAL a chronicle of the latest Nursing News, Practical Articles of the highest value to those who wish to keep themselves abreast with the times, and brightly written articles from Nurses all over the world.

SUBSCRIPTIONS CAN COMMENCE AT ANY TIME.

### TERMS OF SUBSCRIPTION.

### WEEKLY ISSUE.

| | Great Britain. | Foreign and Colonial. |
|---|---|---|
| For One Year ... | 10s. 6d. | 15s. 2d. |
| For Six Months | 5s. 3d. | 7s. 7d. |
| For Three Months... | 2s. 8d. | 3s. 10d. |

### MONTHLY PARTS.

| | Post Free to any Part of the World. |
|---|---|
| For One Year ... | 8s. 6d. |
| For Six Months | 4s. 3d. |
| For Three Months ... | 2s. 2d. |

Postal Orders and Cheques should be made payable to "The Scientific Press, Limited."
N.B.—In order to prevent delay, all business communications should be addressed to "THE MANAGER" only, and not to any person individually.

### CHANGE OF ADDRESS.

In event of change of address, notice should be forwarded to the Publishers by the Saturday previous to date of publication, and the old address should also be given.

"THE HOSPITAL" can be obtained of all Booksellers and Newsagents in the United Kingdom, and at all the Railway Stalls, of Messrs. W. H. Smith and Son; and also from the following Agents in the Colonies and Abroad:—
PARIS: Librairie Galignani, 224, Rue de Rivoli.
BERLIN: Messrs. A. Asher and Co.
LEIPSIG: Mr. F. A. Brockhaus.
NEW YORK: The International News Co.
MONTREAL: The Montreal News Co.
TORONTO: The Toronto News Co.
MELBOURNE, SYDNEY, ADELAIDE, and NEW ZEALAND: Messrs. Gordon and Gotch.
INDIA: At all the Railway Bookstalls and Agencies of Messrs. A. H. Wheeler and Co.
SOUTH AFRICA: Messrs. Juta, Cape Town.

PUBLISHING OFFICES: 428, STRAND, LONDON, W.C.

Now Ready. Third and Revised Edition (twenty-fifth thousand), demy 16mo. (suitable for the apron pocket), handsomely bound in cloth boards, 160 pp., price 2s. In handsome leather, gilt, price 2s. 6d., net.

### THE NURSE'S DICTIONARY

### OF MEDICAL TERMS AND NURSING TREATMENT.

**Compiled for the use of Nurses, by HONNOR MORTEN.**

Containing descriptions of the Principal Medical and Nursing Terms and Abbreviations, Instruments, Drugs, Diseases, Accidents, Treatments, Physiological Names, Operations, Foods, Appliances, &c., &c., encountered in the Ward or Sick-room.

"Very useful for reference, and should be at the disposal of every nurse. It comprises not only what its name implies, but a description of the common abbreviations used, of many instruments, drugs, &c."—*Birmingham Medical Review.*

"A small and convenient manual which can be consulted at a moment's notice."—*Queen.*

---

*Just Published, 8th Edition. Greatly enlarged and thoroughly revised. Crown 8vo., cloth gilt,* 3s. 6d.

### NURSING: ITS THEORY AND PRACTICE,

BEING A

### COMPLETE TEXT-BOOK OF MEDICAL, SURGICAL, AND MONTHLY NURSING.

**By PERCY G. LEWIS, M.D., M.R.C.S., L.S.A., &c., &c.**

To this edition have been added three chapters on Monthly Nursing, together with additions to the chapters on Blood Diseases, on Antiseptics, on the Nursing of Children, and on Private Nursing. Under these headings will be found a short account of the new Aseptic Treatment, the Antitoxin and Serum Treatments, and the Treatment by Thyroid Extracts.

These additions, combined with the revision throughout of the text, render this work the best and most complete Text-book on Nursing in the market, and strengthen its position as a Classic in the Training Schools of the World.

"We have read the book with interest and can warmly recommend it as a nursing manual. . . . Full of useful hints and information."—*Birmingham Medical Review.*

---

Just Published. With numerous Illustrations, demy 8vo., blue buckram, gilt, 3s. 6d.

### DIET IN SICKNESS AND IN HEALTH.

### By Mrs. ERNEST HART,

*Formerly Student of the Faculty of Medicine of Paris, and of the London School of Medicine for Women.*

**With an Introduction by Sir HENRY THOMPSON, F.R.C.S., M.B. Lond.**

SIR HENRY THOMPSON, in his Introduction, says: "I do not hesitate to express my opinion that the present volume forms a handbook to the subject, which will not only interest the dietetic student, but offer him, within its modest compass, a more complete epitome thereof than any work which has yet come under my notice."

"The importance of the subject to which Mrs. Ernest Hart has devoted this volume will not be disputed by any practical medical man. As to how Mrs. Ernest Hart has discharged her self-imposed task we prefer to quote from the introduction the words of Sir Henry Thompson, than whom there could be no more competent critic of a subject which he has himself so much illuminated by his admirable essays."—*British Medical Journal.*

"Medical men will find much of the information given very useful in daily practice. The chapters dealing with the principles which underlie scientific dietetics are characterised by common sense, and are replete with practical suggestions and directions. The chapter on the alimentation of diabetics is of special excellence. Not only are the general rules of diet in this morbid condition carefully discussed and explained, but we have a complete scheme of diet with *menus* for a whole week, showing what a vast amount can be done to palliate the irksomeness of the restrictions by a little ingenuity, forethought, and resource. On the whole we endorse Sir Henry Thompson's recommendation of the book as 'supplying an important want in our educational literature.'"—*The Medical Press.*

---

Third and Revised Edition, demy 8vo., 200 pp., profusely illustrated with Copyright Portraits and Drawings, price 2s. 6d.

### HOW TO BECOME A NURSE: AND HOW TO SUCCEED.

A complete guide to the nursing profession for those who wish to become Nurses, and a useful book of Reference for Nurses who have completed their training and seek employment. Compiled by HONNOR MORTEN (Author of "Sketches of Hospital Life," "The Nurse's Dictionary," &c.).

"To those who are frequently appealed to by girls in their teens or by young women of mature years as to the steps they should take to become nurses, **this book of Miss Morten's must prove a perfect godsend.** It deals with the nursing arrangements of most, if not all, the training schools in London and throughout the country, and gives the novice a thorough insight into the duties expected from her. To those entering, or desirous of entering, any branch of the profession, there is no other guide we know of which contains in so small a compass more useful or practical information. We would advise every one aspiring to be a nurse to consult the book before finally deciding what hospital or maternity institution she elects to train in."—*British Medical Journal.*

"A book that I can most cordially recommend to the notice of any woman or girl desiring to enter the ranks of this profession. The style is bright and interesting and the matter excellent. The volume contains all sorts of useful hints most happily conveyed."—*Woman.*

---

LONDON: THE SCIENTIFIC PRESS (LIMITED), 428, STRAND, W.C.

### ROYAL NATIONAL PENSION FUND FOR NURSES.

*President*—**H.R.H. THE PRINCESS OF WALES.**

*Chairman*—WALTER H. BURNS, Esq. *Deputy-Chairman*—HENRY C. BURDETT, Esq.

**PENSIONS. SICKNESS. ACCIDENT.**

### Total Invested Funds, £250,000.

Nurses are invited to join the Fund on account of the substantial and exceptional advantages which it offers them and which *they cannot obtain elsewhere.* The following are the chief points:—

1. **The Fund is Mutual and essentially Co-operative.**
2. **Easy Payment of Premiums.**
   Nurses can pay their premiums monthly or otherwise as best suits their convenience.
3. **The Fund is open to every Nurse.**
   Nurses can assure for Pensions of any amount commencing at any age.
4. **An Investment and Savings Bank.**
   Those entering under the returnable scale can have their premiums returned to them with compound interest, less a small deduction for working expenses. Interest allowed on deposits.
5. **Additions to Pensions.**
   Besides the Pension entered for, substantial additions thereto may be anticipated in the shape of bonuses.
6. **Sickness and Accident Assurance.**
   Policies are issued in connection with Pension policies assuring 10s., 15s., or 20s. a week in cases of incapacity from work through sickness or accident.

**Full particulars to be obtained from THE SECRETARY,**
ROYAL NATIONAL PENSION FUND FOR NURSES,
28, FINSBURY PAVEMENT, LONDON, E.C.

### TO NURSES.

THE

### SUBSTITUTE FOR STAYS.

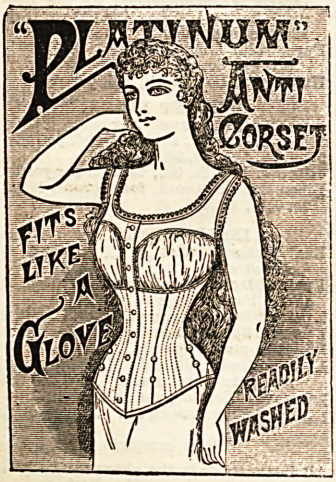

A more Perfect Figure than with ordinary Corsets.

**HIGH AND LOW NECK,**
3/11, 4/11, 6/11, 8/6, &c.
Can also be had with Platinum Busks instead of Buttons, 1s. extra.

Explanatory Pamphlet Free, or Samples sent on approval through a Draper.

LACES DOWN BACK.

**HERTS, SON, and CO., Ltd.,**
WOOD STREET, LONDON, E.C.
Factory—Ridgmount Street, W.C.

### STANDARD TEXT-BOOKS FOR NURSES.

ART OF FEEDING THE INVALID. By a Medical Practitioner and a Lady Professor of Cookery. Demy 8vo. Cloth gilt, 260 pp. 3s. 6d.

A PRACTICAL HANDBOOK OF MIDWIFERY. By FRANCIS W. HAULTAIN, M.D. 18mo. Profusely illustrated. About 260 pp. Handsomely bound in leather. Price 6s.

NURSING. By ISABEL ADAMS HAMPTON. 2nd Edition. Crown 8vo. Cloth, 500 pp. 7s. 6d. net.

SURGICAL WARD-WORK AND NURSING. By ALEXANDER MILES, M.D. (Edin.), C.M., F.R.C.S.E. Demy 8vo. Cloth. With numerous illustrations. 3s. 6d.

HOSPITAL SISTERS AND THEIR DUTIES. By EVA C. E. LUCKES, Matron of the London Hospital. 3rd Edition. Crown 8vo. Cloth. 2s. 6d.

OPHTHALMIC NURSING. By SIDNEY STEPHENSON, M.B., F.R.C.S.E. Crown 8vo. Cloth. Illustrated. 3s. 6d.

MENTAL NURSING. By WILLIAM HARDING, M.D. 2nd Edition. Demy 8vo. Cloth. 2s. 6d.

OUTLINES OF INSANITY. By FRANCIS H. WALMSLEY, M.D. Demy 8vo. Cloth. 3s. 6d. Popular Edition, stitched, 1s. 6d.

ART OF MASSAGE. By A. CREIGHTON HALE. Demy 8vo. Cloth. Illustrated. 6s.

London: THE SCIENTIFIC PRESS (Ltd.), 428, Strand, W.C.

